# Pontocerebellar hypoplasia due to bi-allelic variants in *MINPP1*

**DOI:** 10.1038/s41431-020-00749-x

**Published:** 2020-11-09

**Authors:** Bart Appelhof, Matias Wagner, Julia Hoefele, Anja Heinze, Timo Roser, Margarete Koch-Hogrebe, Stefan D. Roosendaal, Mohammadreza Dehghani, Mohammad Yahya Vahidi Mehrjardi, Erin Torti, Henry Houlden, Reza Maroofian, Farrah Rajabi, Heinrich Sticht, Frank Baas, Dagmar Wieczorek, Rami Abou Jamra

**Affiliations:** 1 Department of Human Genetics, Leiden University Medical Center, Leiden, Netherlands; 2 Institute of Neurogenomics, Helmholtz Zentrum Munich, Neuherberg, Germany, Technical University of Munich, Munich, Germany; 3 Institute of Human Genetics, Klinikum rechts der Isar, School of Medicine, Technical University of Munich, Munich, Germany; 4 Institute of Human Genetics, University Medical Center Leipzig, Leipzig, Germany; 5 Division of Pediatric Neurology, Developmental Medicine and Social Pediatrics, Department of Pediatrics, Dr. von Haunersches Children’s Hospital, Ludwig-Maximilian-University of Munich, Munich, Germany; 6 Vestische Kinder- und Jugendklinik, Datteln, Germany; 7 Department of Radiology, Amsterdam University Medical Centers, Amsterdam, Netherlands; 8 Medical Genetics Research Center, Shahid Sadoughi University of Medical Sciences, Yazd, Iran; 9 GeneDx, Gaithersburg, USA; 10 Department of Neuromuscular Disorders, Queen Square Institute of Neurology, University College London, London, UK; 11 Division of Genetics and Genomics, Boston Children’s Hospital, Boston, Massachussetts, USA; 12 Division of Bioinformatics, Institute of Biochemistry, Friedrich-Alexander -Nürnberg, Erlangen, Germany; 13 Institute of Human Genetics, Medical Faculty, Heinrich-Heine-University Düsseldorf, Düsseldorf, Germany

**Keywords:** Neurodevelopmental disorders, Mutation

## Abstract

Pontocerebellar hypoplasia (PCH) describes a group of rare heterogeneous neurodegenerative diseases with prenatal onset. Here we describe eight children with PCH from four unrelated families harboring the homozygous *MINPP1* (NM_004897.4) variants; c.75_94del, p.(Leu27Argfs*39), c.851 C > A, p.(Ala284Asp), c.1210 C > T, p.(Arg404*), and c.992 T > G, p.(Ile331Ser). The homozygous p.(Leu27Argfs*39) change is predicted to result in a complete absence of MINPP1. The p.(Arg404*) would likely lead to a nonsense mediated decay, or alternatively, a loss of several secondary structure elements impairing protein folding. The missense p.(Ala284Asp) affects a buried, hydrophobic residue within the globular domain. The introduction of aspartic acid is energetically highly unfavorable and therefore predicted to cause a significant reduction in protein stability. The missense p.(Ile331Ser) affects the tight hydrophobic interactions of the isoleucine by the disruption of the polar side chain of serine, destabilizing the structure of MINPP1. The overlap of the above-mentioned genotypes and phenotypes is highly improbable by chance. MINPP1 is the only enzyme that hydrolyses inositol phosphates in the endoplasmic reticulum lumen and several studies support its role in stress induced apoptosis. The pathomechanism explaining the disease mechanism remains unknown, however several others genes of the inositol phosphatase metabolism (e.g., *INPP5K*, *FIG4*, *INPP5E*, *ITPR1*) are correlated with phenotypes of neurodevelopmental disorders. Taken together, we present *MINPP1* as a novel autosomal recessive pontocerebellar hypoplasia gene.

## Introduction

Pontocerebellar hypoplasia (PCH) describes a spectrum of rare genetic neurodegenerative disorders, which are hallmarked by a combination of early atrophy and hypoplasia of the pons and cerebellum. Clinical signs occur within the first months of life and include severe motor and cognitive impairments [1]. Progressive microcephaly is observed and patients often die young. PCH is an extremely rare disease. Hitherto, 13 subtypes have been identified with distinct clinical features or genetic aberration in one of the 19 PCH related genes [2]. Relatively common subtypes are well characterized, i.e., PCH2 (OMIM #277470) is characterized by an invariable pontocerebellar hypoplasia with severe motor and cognitive delay and PCH1 (OMIM # 614678) patients show a PCH2/SMA-like phenotype [3]. However, most subtypes are very rare and only few patients or genotypes are described [4–7]. Still, a molecular diagnosis can be achieved in not all PCH cases. The identification of the causative genetic variant is essential for the counseling of the families and important for the prognosis and recurrence risk.

The mechanisms underlying PCH are not well understood. The initial identification of recessive pathogenic variants in genes encoding tRNA splicing endonuclease (TSEN) complex suggested a link between tRNA processing and cerebellar development (*TSEN54*, *TSEN2*, *TSEN15*, *TSEN34, SEPSECS, RARS2, and CLP1*). However, the identification of other RNA processing genes (*TOE1*, *VRK1*, *EXOSC3*, *EXOSC8*, *EXOSC9*) suggested that the defects are not restricted to tRNA processing and even variants in genes not involved in RNA processing at all may lead to PCH (*CHMP1A*, *TBC1D23*, *PCLO*, *VPS53*, *SLC25A46*, *COASY*) [5, 7–16].

In this study, we describe four different homozygous variants in the multiple inositol polyphosphate phosphatase 1 (*MINPP1*) gene in patients with PCH. The encoded protein MINPP1 (OMIM *605391) is located in the endoplasmic reticulum (ER) lumen where it removes 3-phosphates from inositol substrates [17]. Thus far MINPP1 has not been linked to any human disease.

## Materials and methods

### Ethical approval

Patients were enrolled and sampled according to standard local practice in approved human subjects protocols as part of routine clinical care at the respective Institutes. The project was approved by the Ethic Committee of the University of Leipzig, Germany (224/16-ek and 402/16-ek) and the Technical University Munich, Germany (#5360/12 S) and was conducted in concordance to the declaration of Helsinki. Written informed consent of all examined individuals or their legal representatives for genetic testing and the publication of findings was obtained after advice and information about the risks and benefits of the study. Families were identified via the online match making platform GeneMatcher [18].

### Exome sequencing

For all four families, trio exome sequencing has been performed. As the families come from different centers and have been examined in different time spans, the methods of the exome sequencing differ. Exome capture was carried out with BGI Exome kit capture (59 M), Agilent Sureselect 50 Mb V5 kit, and Nimblegen Seqcap EZ exome enrichment, for families 1, 2, and 3, respectively. Sequencing was performed on a BGISEQ-500, Illumina HiSeq2500 platform, and a SOLiD 5500 platform, respectively. Coverage of at least 10x has been achieved in 98.0%–98.5%, 98.4%–98.7%, and 87.2%–90.0% for the sequenced family members in the families 1, 2, and 3, respectively. Exome sequencing and variant analysis for family 4 was performed as previously described [19].

### Variant prioritization

For family 1, analysis of the raw data was performed using the software Varfeed (Limbus, Rostock) and the variants were annotated and prioritized using the software Varvis (Limbus, Rostock). For family 2 data analysis was done using a custom built bioinformatic pipeline as previously described [20]. For family 3 we performed the analysis using the Ingenuity Variant Analysis™ software (Qiagen, Redwood city).

We have evaluated all annotated variants as well as rare (minor allele frequency below 1%) potential protein-influencing variants in mutation databases (primarily HGMD and ClinVar [21, 22]). We prioritized the variants based on minor allele frequency, inheritance mode, and potential predicted pathogenicity (including in silico values). As no pathogenic or likely pathogenic bi-allelic variants were identified and the families consented for research, we continued evaluation of the sequencing data in a scientific setting in order to identify variants in novel candidate genes. The identified variants were prioritized based on the above mentioned parameters as well as on attributes of the genes, including functional plausibility of the gene, its tolerance for variants (i.e., LOEUF value and Z score [23]), and further aspects (including available animal models, interaction partners, plausibility of the symptoms in regard to the function of the gene).

### 3D modeling of variants

Modeling of the MINPP1 structure was performed with HHpred and Modeler using the structure of Phytase in complex with myo-inositol hexakis sulfate (PDB: 3K4Q) as a template [24–26]. Modeling and evaluation of the variant p.(Ala284Asp) and p.(Ile331Ser) was done with VIPUR [27]. RasMol was used for structure analysis and visualization [28].

## Results

### Clinical description

#### Family 1

The unaffected parents originate from the same small town in Bosnia and have three sons. The older brother is not affected, and the two younger brothers are affected.

**Fig. 1:**
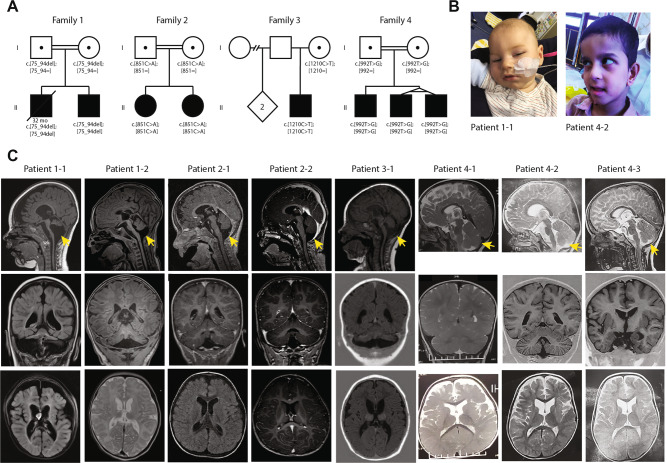
Pedigrees of the families and MRI of the affected children. **a** Pedigrees of the four families showing recessive inheritance pattern. Patient 1-1 is deceased at the age of 32 months. Patient 3-1 received two copies of chromosome 10 from his mother, which lead to an acquired homozygosity. **b** Picture of patient 1-1 and patient 4-2 (**c**) Brain MRI of all eight patients, sagittal (top), coronal (middle) and axial (bottom). The decreased size of the cerebellum is indicated by the yellow arrow. A small pons is seen and on the axial images a decreased size is seen from the caudate nuclei in all patients.

Pregnancy of the male proband (1–1) from family 1 (Fig. 1b) was uneventful. He was born in the 37 + 0 week of gestation (birth weight 2105 g (−2.2 SD), length 44 cm (−2.6 SD), head circumference 31 cm (−2.3 SD)). In the first few months, he showed muscular hypertonia. The first presentation in the clinic was at age of 6 months as he had suffered from seizures. He weighed 5920 g (−1.9 SD), was 60 cm long (−2.7 SD) and showed a remarkable progressive microcephaly with a head circumference of 37 cm (−5.7 SD). Brain MRI revealed a pontocerebellar hypoplasia with a very small pons and reduced volume of the thalamus and basal nuclei. Also, a thin corpus callosum and a dilation of the ventricles and peripheral cerebrospinal fluid (CSF) spaces were observed (Fig. 1C). Severe developmental delay followed and he achieved none of the milestones (no sitting, standing, walking, and speech). Epilepsy was refractory with a permanent seizure activity. EEG showed epilepsy with salaam attacks. Furthermore, he showed stereotypic movements and ataxia. He was severely affected, suffered from recurrent infections and had a percutaneous endoscopic gastrostomy (PEG). He had a micropenis, but no other abnormal clinical findings (no facial dysmorphisms, no abnormalities on ophthalmological examinations and hearing test, apart from possible hyperacusis). He died at the age of 32 months due to an influenza virus infection.

His younger brother (patient 1-2) was born after an uneventful pregnancy at 41 weeks of gestation with a weight of 3645 g (−0.2 SD), length 51 cm (−0.9 SD), and head circumference of 33.5 cm (−1.9 SD). Until the age of 6 months, the parents reported muscular hypertonia and agitation, similar to his brother. At the age of 11 months, his weight was 7100 g (−2.3 SD), length 73 cm (−0.8 SD), and head circumference 38.8 cm (−6.6 SD). He did not have seizures, but presented with significantly delayed development. In addition, he presented stereotypic movements, agitations, stiffness, spasticity, and ataxia. Therefore, he had a markedly similar phenotype to his elder brother before the seizures started. A brain MRI showed similar though milder abnormalities (Fig. 1c). He also has a micropenis, but no other dysmorphologies. Hearing test showed no abnormalities, apart from possible hyperacusis as he startles often and has an impressive Moro reflex. He generally does better than his brother, has no recurrent infections, and does not require tube feeding.

#### Family 2

Patient 2-2 is a girl that was born as second child to consanguineous parents of Turkish origin at term after an uneventful pregnancy (birth weight 4290 g (+2.4 SD), length 54 cm (+1.1 SD), head circumference 36 cm (+1.7 SD)). From early on, she showed a slow development and was able to sit at age 16 months and walked independently 4 months later. She developed secondary microcephaly in the first years. Examination at the age of 8 years revealed generalized hypotonia, ataxia and dysmorphic features with wide nasal bridge, arched eyebrows, up slanting palpebral fissures, epiblepharon, and diastema of the upper incisors. She speaks ~20 words. Brain MRI showed pontocerebellar hypoplasia (see Table 1). The thalami were slightly small, though the basal ganglia were normal. Dilated frontal CSF spaces indicated possible hypoplasia of the frontal lobes (Fig. 1c). She developed bilateral cataracts in toddler age and artificial lenses were inserted at age 3 years. She suffered from refractory seizures since the age of 4 years. EEGs showed continuous generalized slow spike-waves in sleep.

**Table 1:** Clinical findings of patients with *MINPP1* variants.

	family 1 (Dusseldorf/Leipzig)		family 2 (Munich)		family 3 (Leiden)	family 4 (London)			Ucuncu et al.
	patient 1-1	patient 1-2	patient 2-1	patient 2-2	patient 3-1	patient 4-1	patient 4-2	patient 4-3	8 patients
	male	male	female	female	male	male	male	male	5 female/ 3 male
**Variant**									
Genomic Position (hg19) Chr10	g.87504979-87505009del		g.87513139C>A		g.87552224C>T	g.87521094T>G			3 truncating / 4 missense variants
HGVS cDNA (NM_004897.5)	c.75_94del		c.851C>A		c.1210C>T	c.992T>G			
HGVS Protein (NP_055895.1, 1712 aa)	p.(Leu27Argfs*39)		p.(Ala284Asp)		p.(Arg404*)	p.(Ile331Ser)			
GnomAD MAF	0.000008 (2 alleles)		0.00009 (25 alleles)		0.000004 (1 allele)	0 alleles			
Exon	1		3		5	4			
Zygosity	homozygous		homozygous		homozygous	homozygous			7 homozygous / 1 compound heterozygous
**Growth**									
Length/Height	Birth: 44 cm (-2.6 SD) 6 months: 60cm (-2.7 SD)	Birth 51cm (-0.9 SD) 11 months: 73cm (-0.8 SD)	Birth: 50cm (SD) 4 years: 102cm (-0.44 SD)	Birth: 54cm (+1.1 SD) 4 years: 104cm (-0.07 SD)	Birth: n/a 8 months: 77cm (+2.4 SD)	Birth: 49cm (-0.11 SD) 10 years: 110cm (-4.44 SD)	Birth: 42cm (-3.16 SD) 7 years: 109cm (-2.38 SD)	Birth: 45cm (-2 SD) 7 years: 108cm (-2.57 SD)	rather normal birth size with secondary microcephaly
Weight	Birth: 2105g (-2.2 SD) 6 months: 5920g (-1.9 SD)	Birth: 3645g (-0.2 SD) 11 months: 7100g (-2.3 SD)	Birth: 3890g (+1.43 SD) 4 years: 19kg (+0.49 SD)	Birth: 4290g (+2.4 SD) 4 years: 17.8kg (-0.1 SD)	Birth: 4270 (+1.4 SD) 8 month: 9200g (+0.3 SD)	Birth: 2900g (-0.54 SD) 10 years: 17kg (-3.58 SD)	Birth: 2500g (-1.49 SD) 7 years: 12kg (-4.26 SD)	Birth: 2300g (-1.94 SD) 7 years: 13kg (-3.87 SD)	
Head circumference	Birth: 31cm (-2.3 SD) 6months: 37cm (-5.7 SD)	Birth: 33.5cm (-1.9 SD) 11 months: 38.8cm (-6.6 SD)	Birth: 36cm (+1.65 SD) 4 years: 51.5cm (+1.1 SD)	Birth: 36cm (+1.7 SD) 4 years: 47cm (-2.27 SD)	Birth: n/a 8 months: 43.3cm (-1.1 SD)	Birth: 34cm (-0.02 SD) 10 years: 50cm (-2.19 SD)	Birth: 32cm (-1.59 SD) 7 years: 48cm (-3.01 SD)	Birth: 31cm (-2.32 SD) 7 years: 46cm (-4.51 SD)	
**Neurodevelopment**									
Developmental delay/Intellectual disability	yes, severe	yes, severe	yes, severe	yes, severe	yes, moderate	yes, severe	yes, severe	yes, severe	Severe developmental delay with absent milestones
Motor development	delayed	delayed	delayed	delayed	delayed	delayed	delayed	delayed	
Speech	no speech	babbling	delayed	delayed	babbling	no speech	no speech	no speech	
Behaviour	stereotypies, permanently exhausted, salaam movements	stereotypies, agitation	stereotypies	stereotypies	stereotypies, salaam movements	stereotypies, agitation	stereotypies	stereotypies	
Ataxia	yes	yes, with stiffness and spasticity	yes	yes	yes	yes	yes	yes	yes
Epilepsy and EEG	started at age of 6 months, therapy resistant	no	started at age of 4 years, therapy resistant, EEG showed continuous generalized slow spike-waves in sleep	no	started at age of 4 months, therapy resistant, EEG showed hypsarrhythmia	polyspike	started at 6 months, polyspike and wave	started at 6 months, polyspike and wave	7 of 8
Autism	no	no	n/a	yes	no	no	no	no	not indicated
**Cranial MRI**									
Pontine hypoplasia	yes	yes	yes	yes	yes	yes	yes	yes	pontocerebellar hypoplasia in all
Cerebellar hemispheres hypoplasia	yes	yes	yes	yes	yes	no	no	no	
Vermian hypoplasia	yes	yes	yes	yes	yes	yes	yes	yes	
Atrophy of caudate nuclei and putamen	yes	yes, mild	no	no	yes	yes	no	yes	atrophy in all
Thalamic hypoplasia	yes	yes, mild	yes	yes, mild	yes	no	no	no	hypoplasia/atrophy in all
Corpus callosum thinning	yes	no	no	no	yes	yes	yes	yes	thin in 4 from 7
Other white matter abnormalities	yes, delayed myelination	no	no	no	yes	yes (++ frontal)	yes (++ frontal)	yes (++ frontal)	white matter atrophy in 3 of 5
Enlarged ventricles/CSF spaces	yes	yes, mild	yes, mild	no	yes	yes	yes	yes	enlarged in all
Other	global brain atrophy	global brain atrophy	anterotemporal arachnoid cyst	hypoplasia of frontal lobes and insula	-	T2 hyperintensity in basal ganglia	T2 hyperintensity in basal ganglia	T2 hyperintensity in basal ganglia	
Facial Dysmorphism	no	no	n/a	wide nasal bridge	blepharoptosis		low set ear	low set ear	
Hearing	normal, mild hyperacusis	mild hyperacusis, easily scared, impressive Moro reflex	n/a	normal	normal	normal	normal	normal	
Vision	n/a	at least for light	n/a	bilateral cataract, convergent strabismus	nystagmus, possibly cortical blindness	cortical blindness	cortical blindness	cortical blindness	
Other	muscular hypotonia, which started with epilepsy, before that spasticity, micropenis	muscular hypertonia, micropenis	muscular hypotonia cataract on both sides	muscular hypotonia cataract on both sides	hypertonia of extremities, axial hypotonia, micropenis /hypospadias		muscular hypotonia	muscular hypotonia	
Any other variants of interest?	no	no	no	no	AR mutation, described to give micropenis/hypospadias	no	no	no	
Other Notes	PEG, frequent infections of lung, oxygen at home	no infections, no tube feeding	epileptic encephalopathy	epileptic encephalopathy	No PEG	no	no	no	

The patient’s older sister (patient 2-1) has intellectual disability, disruptive behavior disorder and bilateral congenital cataracts. At her last visit at age 4 years, she had no ataxia and a normal head circumference. Brain MRI showed a left temporal arachnoid cyst (size 2.8 × 1.7 × 2.0 cm), and pontocerebellar hypoplasia (Fig. 1c). Like her sister possible atrophy of the frontal lobes indicated by the dilated peripheral CSF space was noticed. The ventricles were mildly dilated. EEG and cardiac ultrasound were normal, as well as metabolic studies.

#### Family 3

Patient 3-1 is a boy who was born to non-consanguineous parents of whom the mother is of Serbian and the father of Dutch origin. He was born at 41 weeks of gestation following an uneventful pregnancy (birth weight 4270 g (+1.4 SD), length and head circumference were not assessed). The boy was born with a micropenis with palpable testis and a bilateral congenital blepharoptosis. No other malformations were seen. At two months the blepharoptosis was surgically corrected and a lack of eye contact was noted. At four months epileptic seizures with salaam attacks occurred with a frequency of four to five times per day. These could have been present since birth and are treated with Mogadon. Other symptoms included nystagmus. At the age of 8 months, his weight was 9200 g (+0.3 SD), length 77 cm (+2.4 SD), and head circumference 43.3 cm (−1.1 SD). He suffered from therapy refractory epilepsy and severe axial hypotonia with hypertonia of the extremities. Feeding was unremarkable and no PEG was needed. Brain MRI at age of five months showed pontocerebellar hypoplasia, with a very small pons and very small basal nuclei and thalamus (Fig. 1c). The corpus callosum was thin and there was possibly white matter atrophy. Also, the ventricles were severely dilated. EEG showed hypsarrhythmia.

#### Family 4

Patient 4-1 is the first-born to consanguineous Iranian parents. He was born at term after an unremarkable pregnancy. His birth weight was 2900 g (−0.54 SD), length 49 cm (−0.11 SD), and head circumference 34 cm (−0.02 SD). Since the first years of life, ataxia and hypotonia were observed. The patient had profound intellectual disability, he was nonverbal and unable to sit by the age of 10 years. He was also diagnosed with cortical blindness. Physical examination revealed axial hypotonia and appendicular spasticity, with a very restricted range of voluntary movements. Brain MRI revealed atrophy of the vermis and pons with sparing of the cerebellar hemispheres (Fig. 1c), T2-weighted hyperintensity of the basal ganglia, atrophy of the dorsal striatum, enlarged ventricles, and severe frontal white matter atrophy. EEG showed a polyspike pattern.

The two younger monozygotic twin brothers of Patient 4-1 (Patients 4-2 and 4-3, Fig.1b) were similarly affected. They were born at 37 weeks’ gestation after an unremarkable pregnancy. Birth weights were 2500 g (−1.49 SD) and 2300 g (−1.94 SD), lengths 42 cm (−3.16 SD) and 45 cm (−2 SD), and head circumferences 32 cm (−1.59 SD) and 31 cm (−2.32 SD) for Patients 4-2 and 4-3, respectively. Both children were diagnosed with severe intellectual disability, ataxia and cortical blindness. At the age of 6 months, they started to suffer from epileptic seizures which only partially responded to antiepileptic treatment, with a frequency of once a month. At the age of 7 years, both children were nonverbal and unable to sit. Physical examination revealed severe axial hypotonia and spastic tetraplegia. Neuroimaging findings were very similar to those observed in the older brother, except for a sparing of the putamen and caudate nucleus in patient 4-2. EEG revealed a polyspike and wave pattern in both twins.

### Genetic results

**Fig. 2:**
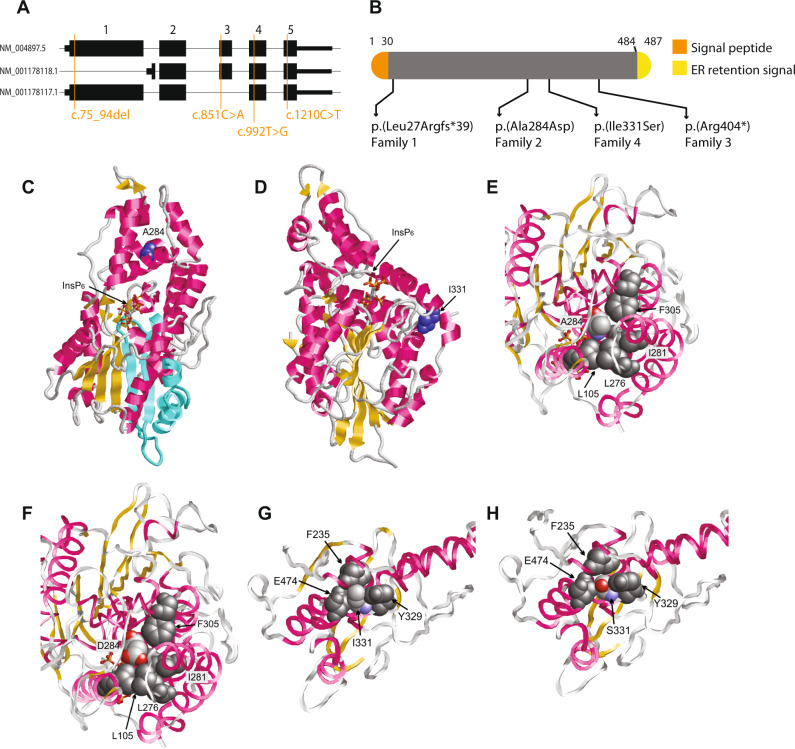
Molecular modeling. Locations of the variants in MINPP1 gene (**a**) and protein (**b**). Model of the MINPP1 structure (**c**–**h**) illustrating the effect of truncation and missense variants and the location of the variants on the gene. **c** Structure of MINPP1 (backbone representation) showing the location of p.A284 (blue) and the bound InsP_6_ substrate (stick representation). Also, the p.Arg404* truncation variant causes the loss of several elements in secondary structure (missing elements are indicated in cyan). **d** Structure of MINPP1 (backbone representation) showing the location of p.Iso331 (blue) and the bound InsP_6_ substrate (stick representation). **e** p.Ala284 is buried in the hydrophobic core of MINPP1. p.Ala284 is colored according to the atom types and the interacting hydrophobic residues are shown in dark gray. **f** In the p.Ala284Asp variant a charged carboxyl group is placed within the hydrophobic protein core (color coding as in (**e**)). **f** and **g** p.Iso331 forms tight hydrophobic interactions with the aromatic residues p.Phe235 and p.Tyr329, and with the hydrophobic methylene groups of the p.Glu474 sidechain. p.Iso331 is colored according to the atom types and the interacting residues are shown in dark gray. **h** In the p.Iso331Ser variant the smaller polar serine sidechain is placed within the hydrophobic environment leading to a destabilization of the MINPP1 structure (color coding as in (**g**)).

Whole Exome Sequencing revealed exonic variants in *MINPP1* (NM_004897.5) in all patients (Fig. 2a). In both affected boys of family 1 a homozygous frameshift variant c.75_94del, p.(Leu27Argfs*39) was identified, their parents were heterozygous carrier for this variant. In family 2, a homozygous missense variant c.[851 C > A], p.(Ala284Asp) was identified in both affected sisters, their parents were also heterozygous carrier. In family 3, a homozygous nonsense variant c.1210 C > T, p.(Arg404*) was identified in the patient. The mother was heterozygous and the father did not carry the variant. Further inspection of the data revealed a maternal isodisomy of chromosome 10 which was confirmed by SNP array analysis. In the three siblings from family 4 the homozygous missense variant c.992 T > G, p.(Ile331Ser) was identified.

The variant detected in family 1 was reported in a heterozygous state in two persons in gnomAD (MAF of 0.000008 [23],). The variant of family 2 was present in 25 alleles (MAF of 0.00009) of the gnomAD database but never reported in homozygous state. The variant of family 3 was present in gnomAD with one allele (MAF of 0.000004). The variant from family 4 has not been reported before.

### Structural analysis

Since cell lines of the patients were not available for functional analysis, we mapped the identified predicted protein changes on the MINPP1 structure. The frameshift caused by the variant p.(Leu27Argfs*39) is located within the MINPP1 signal peptide that spans residues 1-30 (Fig. 2b). Therefore, this variant is expected to result in the complete absence of MINPP1. 3D modeling of the protein reveals that the MINPP1 adopts a well-defined three-dimensional structure that also harbors the central ligand binding site (Fig. 2c). The variant c.1210 C > T, predicted to result in p.(Arg404*), identified in family 3 would possibly lead to nonsense mediated decay. However, because the c.1210 C > T variant is located in the last exon of *MINPP1*, a truncated protein (p.(Arg404*)) could be translated, but would result in loss of several secondary structure elements (Fig. 2c). This would likely cause unfolding of the protein and a loss in enzymatic activity. The p.(Ala284Asp) missense substitution affects a residue that is buried within the globular domain of MINPP1 (Fig. 2c). A closer inspection of the structure shows that A284 forms hydrophobic interactions with the adjacent residues L105, L276, I281, and F305 (Fig. 2e). In the A284D variant a charged carboxyl group is placed within the hydrophobic core (Fig. 2f), which is energetically highly unfavorable and therefore predicted to cause a significant reduction in protein stability and enzymatic activity. As to the p.(Ile331Ser) missense substitution, the I331 interacts with the hydrophobic methylene groups of the p.E474 sidechain and with aromatic residues p.F235 and p.Y329. The p.(Ile331Ser) substitution leads to the placement of a hydrophilic side chain in the hydrophobic environment. Therefore, it is likely that the p.(Ile331Ser) substitution disrupts the protein structure.

## Discussion

Here we describe eight children from four unrelated families with development delay and a specific phenotype overlapping PCH (Table 1 and Fig. 1). Overlapping clinical signs include microcephaly or smaller than average head circumference, epilepsy, micropenis, cataract, ataxia, muscular hyper- and hypotonia, and stereotypies (see Table 1 for further details).

Exome sequencing revealed in all eight affected individuals homozygous, seemingly pathogenic variants in *MINPP1*. In patients 1-1, 1-2, and 3-1 from families 1 and 3, homozygous truncating variants were identified and are most probably pathogenic. In patients 2-1 2-2, 4-1, 4-2 and 4-3 from family 2 and 4 two homozygous missense variants assumed to be deleterious based on structural analysis were identified.

The minor allele frequency of all *MINPP1* loss of function (LoF) variants in gnomAD is 1:6500 and there are no homozygous LoF. Assuming that the first identified LoF in this study is not relevant to the phenotype, the probability of identifying a second homozygous LoF in a cohort of 50,000 cases (there are 44,000 entries in GeneMatcher) by chance is 0.0082, making a coincidental event highly improbable.

The above-mentioned lines of evidence let us conclude that *MINPP1* is a strong candidate gene for syndromic pontocerebellar hypoplasia due to bi-allelic LoF variants.

The three male patients from families 1 and 3 presented with a micropenis. However, the two sisters of family 2 and the three male patients from family 4 did not show a disorder of genitalia. At least two neurological disorders with PCH have already been observed in combination with a disorder of sexual development: PCH7 due to bi-allelic variants in *TOE1* (OMIM # 614969, *613931 [9]) and Joubert syndrome 1 due to bi-allelic variants in *INPP5E* (OMIM # 213300, *613037). Thus, a disorder of sexual development may possibly be a part of the clinical spectrum of *MINPP1*, but the number of cases is too small to draw a definite conclusion. Furthermore, in family 3 we identified a variant in the androgen receptor gene *AR* (OMIM *313700) that may lead to the androgen insensitivity syndrome (OMIM # 300068) and thus explain the micropenis regardless of *MINPP1*.

During submission of this manuscript, a preprint server publication describing eight other PCH patients with bi-allelic *MINPP1* variants became available [29]. The symptoms were comparable such as having, in addition to the clear pontocerebellar hypoplasia, secondary microcephaly, ataxia, spasticity, severe developmental delay, epilepsy, vision issues (cataract, blindness, and others). Compared to other PCH subtypes (e.g., PCH1B-C or PCH2A), MINPP1 patients live longer and have slightly milder symptoms. Brain MRI indicates that the cerebellar hemispheres are often only mildly affected. Also, white matter and frontal cortex atrophy, and the involvement of the basal ganglia could be an indicator of MINPP1 related PCH.

How *MINPP1* aberrations result in a PCH phenotype remains enigmatic. According to the literature, inositol polyphosphates (InsPs) were shown to play signaling roles in the regulation of calcium mobilization, cell growth, vesicular transport, gene expression, and export of mRNA and apoptosis [30–34]. MINPP1 is an ER luminal soluble protein and dephosphorylates InsPs by removing specifically phosphate group at position 3 and association with apoptosis has been described extensively [17, 35–41]. MINPP1 is the only ER resident enzyme that can hydrolyze inositol pentakisphosphate and inositol hexakisphosphate and it exhibits characteristics of a stress-responsive molecule during ER stress-induced apoptosis regardless of the underlying involved mechanisms [42]. Accordingly, the deregulation of apoptosis might be a possible pathomechanism in our patients as affected children also showed enlarged CSF areas suggestive of brain atrophy. However, this still need more research to be understood. Initially, *MINPP1* was not a convincing candidate gene for PCH as Minpp1-deficient mice are viable, fertile, and without obvious defects [43]. However, the identification of four families with comparable MINPP1 aberrations and a specific overlapping phenotype make it a very consistent candidate gene. Thus, we suggest re-analyzing the mouse model regarding neurological phenotypes.

An interactor with MINPP1 substrates is the inositol, 1,4,5 triphosphate receptor type 1 (ITPR1). Upon binding IP3, ITRP1 regulates Ca2+ release from the ER. Bi-allelic recessive pathogenic variants in *ITPR1* are reported to cause PCH-like phenotype. Also, other genes encoding proteins of the inositol phosphatase metabolism (e.g., *INPP5K, FIG4, INPP5E*) are associated with autosomal recessive syndromes of intellectual disability [44–47]. It seems therefor plausible that loss of the MINPP1 enzymatic activity would lead to a comparable disorder. Until now, proteins involved in metabolic steps pre- or post- MINPP1 (e.g., ITPKA, ITPKB, ITPKC, ITPK1, and IPKM) are not associated with phenotypes. However, considering the above-mentioned arguments, it is not unlikely that they will be linked to human disease in the next future.

Although we still do not have clear functional proof of causality between *MINPP1* variants identified in four families and the observed clinical phenotype, based on the noncoincidental overlap of features and genotypes of the affected children as well as the relevance of the inositol phosphatase metabolism to neurological disorders (including PCH), we conclude that sequence variants in *MINPP1* are associated with a new form of syndromic pontocerebellar hypoplasia.

## Note

After submission of this paper, a fifth family with a homozygous MINPP1 frameshift variant c.1401delG p.(Ser468Valfs*10) in MINPP1 and similar symptoms was identified. Patient 5-1 presented at 12 months of age with global developmental delay, brain malformation, microcephaly, epilepsy, feeding difficulty, laryngomalacia, and hypogonadism. He was full born to consanguineous parents. The pregnancy was unremarkable (weight 3.12 kg). He developed seizures at 4 months of age. The patient had multiple episodes of aspiration pneumonia and transitioned to nasogastric feeding. At 12 months he presented with hypopigmented oval macula and severe microcephaly (head circumference 41 cm, −4.46 SD) and an upturned nose. His global motor development was delayed and vision was low. Brain MRI at 16 months of age showed structural abnormalities of the posterior fossa including marked hypoplasia of the brainstem, cerebellar hemispheres and cerebella peduncles. He had signal abnormality of the deep gray matter and hippocampi, posterior predominant periventricular white matter volume loss, thinning of the posterior corpus callosum, and signal abnormality within the bilateral cerebellar hemispheres
